# Mechanisms and effects of NLRP3 in digestive cancers

**DOI:** 10.1038/s41420-023-01783-6

**Published:** 2024-01-05

**Authors:** Yuxin Si, Lei Liu, Zhe Fan

**Affiliations:** 1 Department of General Surgery, The Third People’s Hospital of Dalian, Dalian Medical University, Dalian, China; 2 Department of General Surgery, The Second Affiliated Hospital of Dalian Medical University, Dalian, China; 3 Laboratory of Pathogenic Biology, College of Basic Medical Science, Dalian Medical University, Dalian, China

**Keywords:** Cancer, Cancer

## Abstract

Inflammasomes are thought to be important mediators of host defense against microbial pathogens and maintenance of gastrointestinal tract homeostasis. They can modulate caspase-1 to promote IL-18 and IL-1β secretion and promote phagocytosis induced by bacterial pathogens. NLRP3 is an inflammasome comprising a multiprotein complex assembled by pattern recognition receptors in the cell cytoplasm. It is a crucial component of the innate immune system. Dysregulation of NLRP3 may contribute to inflammatory diseases and intestinal cancers. Recent research suggests that NLRP3 plays an essential role in tumor development; therefore, intensive study of its mechanism is warranted as it could play a key role in the treatment of digestive system tumors. In this review, we discuss the mechanism and role of NLRP3 in tumors of the digestive system and response strategies to modulate NLRP3 for potential use in tumor treatment.

## Facts

- Effects of NLRP3 in colorectal cancer.
- Effects of NLRP3 in gastric cancer.
- Effects of NLRP3 in liver cancer.
- Effects of NLRP3 in pancreatic cancer.

## Open Questions

- What are the characters of NLR families?
- What are the molecular processes in the digestive cancers process of NLRP3?
- What are the effects for NLRP3 in different kinds of cancer?

## Introduction

According to the latest data from the Global Cancer Database, the digestive system is the source of 1.5 of the 3 million deaths from cancer in China each year [1]. Digestive system tumors account for 50% of cancer mortality in China. From the esophagus to the anus, tumors may appear in any part of the gastrointestinal (GI) tract. Esophageal, gastric, and colorectal cancers rank third, fourth, and fifth, respectively, in incidence and mortality of malignant tumors in China, and together account for half of all common malignant tumors. Inflammation is a potential risk factor for cancer occurrence, with all stages of carcinogenesis influenced by inflammation. Therefore, maintaining intestinal homeostasis and balancing inflammation are essential to prevent the development of GI cancers.

The activation of inflammasomes is an important signaling mechanism leading to acute and chronic inflammation [2–4]. Inflammasomes are multi-protein complexes participating in the assembly of intracytoplasmic pattern recognition receptors (PRRS). They are a crucial part of the innate immune system. Inflammasomes recognize pathogen-associated molecular patterns (PAMPs) or host-derived danger signaling molecules (DAMPs) to raise and stimulate the pro-inflammatory protease Caspase-1. Activated Caspase-1 cleaves precursors of interleukin (IL)-1β and IL-18 and produces the corresponding mature cell factor [5–7]. Five kinds of inflammasomes have been identified: NLRP1, NLRP3, NLRC4, IPAF, and AIM2. The known inflammasomes typically contain apoptosis-related microprotein, caspase proteases, and a NOD-like receptor family protein or HIN200 family protein. Table 1.

**Table 1:** Different roles of NLRP3 inflammatory body in tumor occurrence and development.

	Mechanism of inhibiting tumor occurrence and development	Mechanism of promoting tumor occurrence and development
**Colorectal cancer**	NLRP3 mediates STING signal transduction of macrophages, which promotes anti-tumor characteristics of 4-1BBL/4-1BBL-dependent NK cells and thus inhibits cancer development. NLRP3 mediates IFN-g to activate tumor suppressor STAT1, which has a protective effect on tumorigenesis.	NLRP3 is activated by the crosstalk of mφ-CRC cells, which leads to faster migration of cancer cells. Overproduction of 5-HT/TPH1 promotes cancer progression by enhancing inflammasome activation of NLRP3. NLRP3 can promote the proliferation and metastasis by regulating epithelial–mesenchymal transition.
**Gastric cancer**		HP/M.hy leads to the activation of NLRP3 inflammatory corpuscle pathway and results in the migration and invasion of gastric cancer cells. NLRP3 is combined with cyclin-D1 promoter, which promotes its transcription, enhances proliferation and GC occurrence. CagA can promote the invasion and migration of gastric cancer cells by activating NLRP3 inflammatory corpuscles and generating ROS in cells.
**Liver cancer**	ROS mediate the inflammatory corpuscles of NLRP3 and inhibit the NF-kB signaling pathway in hepatocellular carcinoma cells, thus protecting tumor occurrence. The expression of NLRP3 inflammatory corpuscles in liver cancer tissue decreased significantly and was negatively correlated with the pathological grade and clinical stage of liver cancer patients, so NLRP3 has a protective effect in cancer development.	Obesity-induced ER stress will lead to focal death of liver NLRP3 inflammatory corpuscles, thus mediating liver injury.
**Pancreatic cancer**		Platelet NLRP3 signal transduction promotes platelet activation and aggregation in PDA, thus promoting tumor growth. NLRP3 deficiency inhibits the growth and progression of PDA tumor and cut down the occurrence of lung metastasis. On the contrary, the activation of NLRP3 enhanced the lung metastasis of pancreatic ductal adenocarcinoma.

The NLRP3 inflammasome, an integral part of innate immunity, plays a crucial role in both the immune response and disease development. NLRP3 is one of the most multifunctional members of the NLR family. It can regulate the immune system, apoptosis, cell growth, and gut microbiome, thereby influencing cancer development [8]. Due to their ability to be activated by various types of pathogens or danger signals, NLRP3 inflammasomes play a crucial role in various disease processes, including initially confirmed familial periodic autoinflammatory reaction, type 2 diabetes, Alzheimer’s disease, and atherosclerosis [9]. Thus, as a crucial aspect of the inflammatory reaction, the NLRP3 inflammasome can potentially also provide new treatment targets for various inflammatory diseases.

**Fig. 1:**
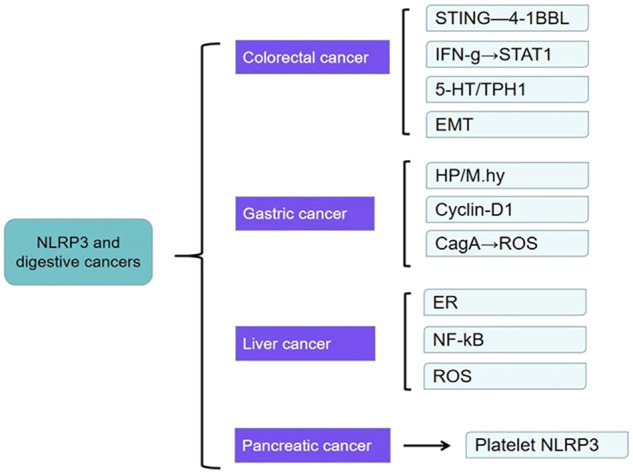
Mechanisms of NLRP3 in common digestive tract cancers.

This review focuses on a collection of recent evidence focusing on the function of NLRP3 in the occurrence and development of digestive cancers (DCs) and its activation and inactivation pathways. Additionally, we present some potential uses of this inflammasome as a therapeutic target for treating DC (Fig. 1).

### NLRP3 and colorectal cancer

Colorectal cancer (CRC) is a common digestive system malignant tumor, which involves the transformation of regular epithelial cells to adenomas and then to cancers or the transformation of chronically inflamed tissue (colitis) to cancer. Although its incidence and mortality are lower than those of gastric, esophageal, and primary liver cancer, at present, globally, it is a common digestive cancer. Its incidence has been increasing over the past several years [10]. The influence of NLRP3 on CRC is still unclear. It seems to have a protective function, although it may have the opposite effect. It has been shown that NLRP3-mediated activation of interleukin (IL)-18 and IL-1β is responsible for macrophage stimulation of interferon genes’ (STING) signaling and the promotion of 4-1BBL/4-1BBL-dependent natural killer (NK) cell anti-tumor properties thereby inhibiting the metastatic growth of CRC in the liver [11, 12]. It has also been suggested that the NLRP3 inflammasome can activate the tumor suppressor STAT1 in the colon through IL-18-mediated interferon gamma (IFN-ɣ) production. Thus it has a protective effect against colitis-related tumorigenesis [13].

**Fig. 2:**
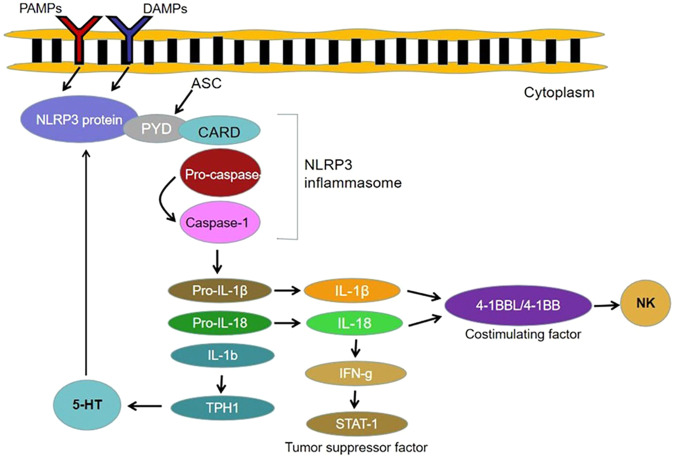
Schematic diagram of the mechanism of NLRP3 in the development of colorectal cancer.

On the contrary, one study found that NLRP3 inflammasomes can be activated by MΦ-CRC cell crosstalk in macrophages, which leads to faster migration of CRC cells and blockage of NLRP3 signal transduction. Thus it inhibits the migration and metastasis of CRC cells in vitro and in vivo, revealing that NLRP3 signal activation can stimulate the migration and invasion of CRC cells in macrophages [14]. Du et al. found that a high cholesterol diet can cause a chronic auto-inflammatory response that can lead to serious health problems, including CRC [15]. In their experimental data, dietary cholesterol significantly increased the inflammatory response and tumor burden by stimulating the activation of NLRP3 inflammasomes in macrophages, thereby promoting oxidative azomethanes (AOM)-induced CRC. In contrast to the normal colonic epithelium, the level of 5-HT and the expression of 5-HT biosynthesis restriction enzyme tryptophan hydroxylase 1 (TPH1) were significantly increased in colonic tissue from CRC patients. Excessive production of 5-HT in the gastrointestinal tract promotes the progression of rectal cancer through the enhancement of inflammasome-mediated activation of NLRP3 (Fig. 2) [16].

**Fig. 3:**
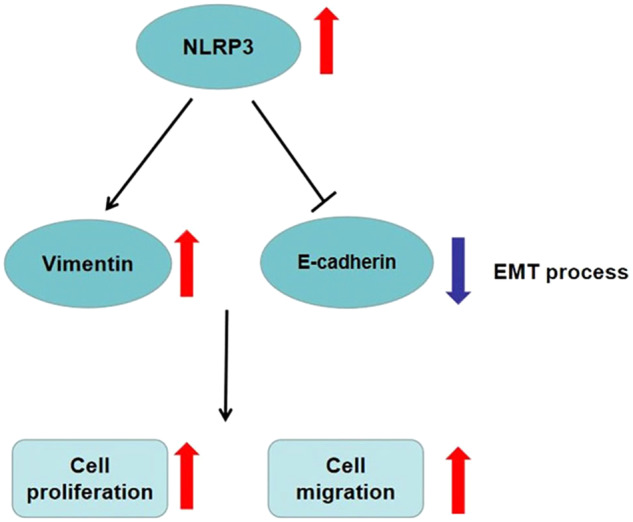
NLRP3 regulates epithelial–mesenchymal transition to promote proliferation and metastasis of colorectal cancer cells.

Some studies have established nude mouse CRC xenografts by detecting the expression of NLRP3 in CRC patients and subcutaneously injecting HCT116 or RKO cells. Cell proliferation and apoptosis were observed after treating HCT116 cells with NLRP3 inhibitor MCC950 [17]. It was found that high NLRP3 level are correlated with tumor staging, distant metastasis, and vascular invasion of malignant tumors. Additionally, high NLRP3 levels correlate with a low 5-year survival rate and even lower 10-year survival rate. Conversely, low levels of NLRP3 expression correlate with a more favorable prognosis for CRC [17]. It has also been shown that NLRP3 can stimulate proliferation and metastasis of CRC cells by adjusting epithelial interstitial transformation (Fig. 3) [18, 19]. Wang et al. described Porphyromonas gingivalis promotion of CRC through the activation of hematopoietic NLRP3 inflammasomes [20].

Glutathione transferase Omega-1 (GSTO1) can prevent azomethane induced colorectal tumor formation through Omega like glutathione transferase GSTO1-1 regulation of the pro-inflammatory cytokine IL-1 by removing the protective cellular mechanisms of NEK7 in NLRP3 inflammasomes, stimulation of β release of IL-18 and protection against CRC formation. Although GSTO1-1 is a new target for regulating cytokines IL-1β and IL-18 related to NLRP3 inflammasomes through small molecular inhibitors, in some cases anti-inflammatory drugs targeting these cytokines may increase the risk of CRC occurrence [21].

Chronic inflammation is an important driving factor for colorectal carcinomatosis. 5-HT has been reported to be a neurotransmitter that may boost gastrointestinal inflammation. It has been found that NLRP3 inflammasomes mediate IL-1b maturation and upregulate the biosynthesis of 5-HT in CRC cells by leading TPH1 transcription, bringing the positive feedback pathway between 5-HT and NLRP3 signal transduction to light [16]. The potential therapeutic target of CRC would involve the positive feedback route between 5-HT and NLRP3 signals. Increasing amounts of evidence demonstrate that gut flora dysregulation plays a vital role in the development of colitis-related cancer, suggesting that targeted intervention with gut flora as well as its metabolic products may be potential treatment targets. It has been noted that Bacteroides fragilis numbers are significantly reduced during the development of colitis-associated cancers (CAC) [22]. It has been experimentally demonstrated that broad-spectrum antibiotic (BSAB) intervention in the enteric microbiome of mice accelerates the development of cancer. However, gastric transplantation of B. fragilis is able counteract the effects of BSAB intervention. The mechanism is B. fragilis mediated promotion of the secretion of short-chain fatty acids (SCFAs) in the intestine. Then, SCFAs (especially butyrate) negatively regulate the NLRP3-mediated inflammatory signaling route, inhibiting macrophage activation and secretion of mediators such as IL-18 and IL-1β, reducing the level of intestinal inflammation, limiting the development of CAC. A study demonstrated that B. fragilis colonization can effectively improve intestinal epithelial injury caused by chronic inflammation and prevent CRC tumor expansion, which is a treatment method strategy with good prospects for clinical application [22].

### NLRP3 and gastric cancer

**Fig. 4:**
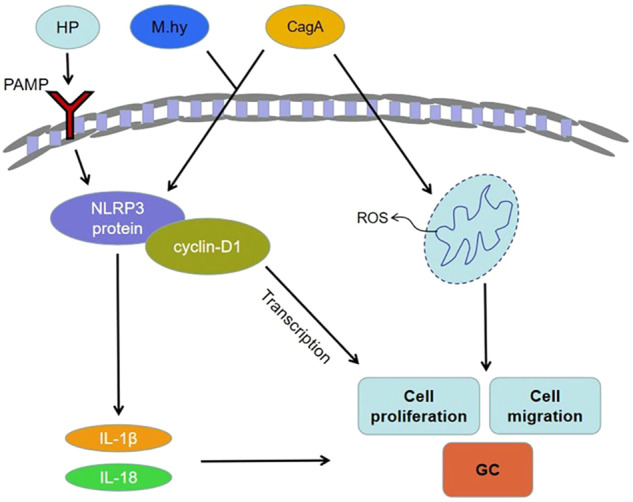
Schematic diagram of the mechanism of NLRP3 in the development of gastric cancer.

Gastric cancer (GC) is a malignancy of the gastric mucosal epithelium. Although gastric cancers can occur anywhere in the stomach, more than half occur in the gastric antrum. The greater and lesser curvatures of the stomach and the front and rear walls can also be involved. Most gastric cancers are adenocarcinomas. The potential role of NLRP3 in GC is not well understood. A large number of research reports focusing on GC and NLRP3 are related to cancer secondary to gastritis caused by Helicobacter pylori. Activation of PAMP, the NLRP3 inflammatory vesicle pathway, and production of IL-1β and IL-18 are due to the release of H. pylori [23–25]. Mycoplasma porcineum can also activate the NLRP3 inflammasome and promote migration and invasion of GC cells [26]. Research has revealed that the content of NLRP3 in GC is significantly increased, which promotes the stimulation of NLRP3 inflammasomes and secretion of IL-1β in macrophages [27]. Furthermore, NLRP3 can combine with the cyclin-D1 promoter to promote its transcription in gastric epithelial cells, resulting in the enhanced proliferation of epithelial cells and the occurrence of GC. Studies have demonstrated that cytotoxin-associated gene A (CagA) boosts the invasion and migration of GC cells by activating NLRP3 inflammasomes and intracellular reactive oxidative species (ROS) generation [28]. This finding also provides a new view on the mechanism of GC induction by H. pylori. Another study highlighted that NLRP3 has a strong association with immune infiltration and cancer prognosis [29]. The over expression of NLRP1/NLRP3 likely fosters the germination of GC. Therefore high levels of NLRP3 expression may be an underlying risk factor for the invasion and metastasis of GC, which is related to the bad prognosis of GC patients and may be used as a prognostic biomarker of GC (Fig. 4).

The constitutive expression of miR-22 in the gastric mucosa was identified as an inhibitor of NLRP3. MiR-22 directly targets NLRP3 and can weaken its carcinogenic potential in vivo and in vitro. At the same time, H. pylori infection was also found to suppress miR-22 expression while enhancing NLRP3 expression, thereby triggering uncontrolled epithelial cell proliferation and the appearance of GC. One study revealed the mechanism of miR-22 inhibiting NLRP3 and maintaining the gastric microenvironment homeostasis and indicated that miR-22 was the potential GC intervention target [27]. Previously, Icariside (ICA) was found to inhibit GC cell growth by modulating the hsa_circ_0003159/miR-223/nlrp3 signaling axis [30]. ICA also negatively affects the invasion and migration of GC cells, which may provide potential molecular targets and therapeutic agents for its treatment. It has also been suggested that, compared with normal tissues and cells, lncRNA ADAMTS9-AS2 is less expressed in GC cells and tissues. LncRNA ADAMTS9 may regulate NLRP3 inflammasomes in GC cells by targeting miR-223-3p to inhibit GC development [31]. Much evidence has shown that IL-1β is related to the development of gastric cancer. Therefore, downregulating the production of IL-1β mediated by Helicobacter pylori may be a way to prevent gastric cancer. Kim et al. showed that Withaferin A, a purified withanolide from withania somnifera with anti-inflammatory and anti-tumor effects, could inhibit the production of IL-1β by Helicobacter pylori in dendritic cells and could be used as a novel drug for GC prevention and treatment [32].

### NLRP3 and liver cancer

The most common primary malignant tumor of the liver is hepatocellular carcinoma (HCC) which originates from the epithelial or mesenchymal tissues of the liver. It is a highly dangerous malignancy with a relatively high-incidence and it is also the third leading cause of cancer death globally. The etiology and exact molecular mechanism of primary liver cancer are not completely clear. It is understood that its pathogenesis is very complex, influenced by both environment and diet.

The role of NLRP3 in liver cancer is unclear. Liver fibrosis is found in the early phase of various liver diseases. If it cannot be controlled opportunely, liver fibrosis will develop into cirrhosis, hepatic failure, and/or even hepatocarcinoma. In fact, the vast majority of hepatocellular carcinomas are secondary to chronic hepatitis or cirrhosis. Evidence suggests that autophagy and NLRP3 inflammasomes play an important role in liver fibrosis [33]. One study analyzed the expression of NLRP3 in normal liver tissue and liver tissue from patients with hepatitis, cirrhosis, and hepatocellular carcinoma, representing different stages of hepatocarcinogenesis [34]. Immunohistochemical staining patterns of NLRP3 inflammatory vesicle components showed a significant decrease in NLRP3 inflammatory vesicle component expression in HCC tissue compared to surrounding hepatitis and cirrhotic tissue; however, there were no significant differences between normal and HCC tissue. The data show that the expression of NLRP3 inflammasomes in normal liver tissues is low, but it is upregulated in liver injury (i.e. hepatitis and cirrhosis) [35]. Compared to non-cancerous liver tissue, the expression of NLRP3 inflammasomes in HCC tissue was significantly decreased. The NLRP3 inflammasome expression levels also showed a negative correlation with pathological grade and clinical stage in patients with liver cancer. Additionally, the expression of the inflammasome component of NLRP3 in patients with advanced liver cancer was even lower. These data all suggest a protective role of NLRP3 inflammasomes in cancer progression in addition to their recognized roles as immune and inflammatory inducers.

**Fig. 5:**
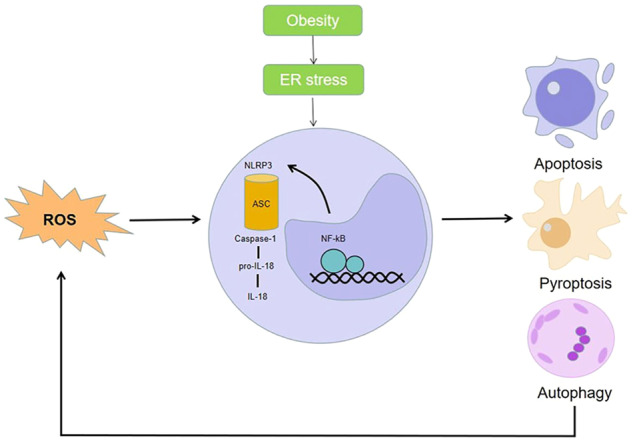
Schematic diagram of the mechanism of NLRP3 in hepatocellular carcinoma.

In addition, it has been reported that activation of NLRP3 inflammasomes plays an important role in obesity-mediated metabolic disorders [36]. Due to the increasing prevalence of obesity in recent years, the incidence of chronic liver disease is rising. This will induce endoplasmic reticulum (ER) stress, leading to molecular death and inflammation. In a study by Lebeaupin et al., the interactions between hepatic ER stress, NLRP3 inflammatory vesicle activation, and stem cell death signaling were explored [37]. Also, Wei et al. were able to elucidate the link between endoplasmic reticulum stress signaling and HCC pathogenesis [38]. Fatty degeneration is a common disease that is found in obese patients. Twenty five percent of these patients will develop steatohepatitis and, therefore, with a risk of developing liver cancer. Researchers analyzed human steatohepatitis biopsies to show a correlation between elevated inflammasomes and ER stress markers and liver injury, revealing that ER stress can lead to focal death of inflammasomes of liver NLRP3, thus mediating liver injury. Inhibiting the activation of ER-dependent inflammasomes and cell death may be a potential treatment for chronic liver disease (Fig. 5).

Scopolamine (ANI), an alkaloid derived from scopolamine, has good therapeutic effects on infectious shock and morphine addiction, as well as significant anti-inflammatory effects; however, the role of ANI in hepatocellular carcinoma has rarely been investigated. Research by Li et al. determined that the anticancer effects of scopolamine could inhibit HCC cell growth and motility, induce apoptosis, and maintain Th1/Th2 balance by suppressing NLRP3 inflammatory vesicle expression and thus NLRP3 inflammatory vesicle activation [39]. Thus, ANI may be a promising potential therapeutic agent for HCC.

Hepatic fibrosis is a pathophysiological process, which caused by an abnormal proliferation of connective tissue in the liver caused by various factors. A degree of liver injury will occur in the process of liver repair. If the tissue is exposed to damaging factors for a long time, the process of fibrosis will develop into cirrhosis and then may develop into liver cancer. Some studies have shown that autophagy and NLRP3 inflammasomes have a significant effect on the process of liver fibrosis. As the main lower reaches signal NLRP3 inflammasome transduction, the IL-1 family route is an important medium for liver damage and fibrosis [33, 40]. Therefore, the modulation of autophagy and NLRP3 inflammasomes may be defined as a treatment target for liver fibrosis. The results of these studies need further clinical trials for verification.

Dai et al. [41], found that the folk remedy Shuang Hua Tang (SHD) had an inhibitory effect on HCC by a mechanism that exerted an anticancer effect by activating ROS-mediated NLRP3 inflammasomes and repressing the NF-kB signaling route in liver cancer cells [42]. It has also been shown that carotenosterol can attenuate alcohol-induced liver injury and inflammation via the P38/NF-kB/NLRP3 inflammatory vesicle pathway [43]. Overall, this study and TCM theoretical and pharmacological studies suggest that Shuang Hua Tang may be a promising herbal medicine for the treatment of liver cancer. It was also revealed in a study that miRNA-223-3p promotes apoptosis and inhibits proliferation of human hepatocellular carcinoma cells (hep3B) by regulating NLRP3, and miR-233 represses the proliferation of HCC cells and promotes apoptosis by directly targeting NLRP3 [44].

### NLRP3 and pancreatic cancer

**Fig. 6:**
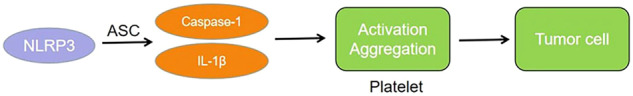
Schematic diagram of the role of platelet NLRP3 in pancreatic cancer.

Pancreatic cancer (PDA), another malignant tumor of the digestive system, is also known as “the king of cancer”. According to *The Lancet*, the 5-year survival rate of PDA is only 10%, making it one of the most fatal malignant tumors [45]. PDA’s insidious nature and atypical clinical symptoms make it difficult to diagnose and treat. Its incidence and mortality have increased significantly in recent years. The cause of PDA is undefined. However, associations with smoking, alcohol consumption, eating high-fat foods, drinking too much coffee, environmental damage, and hereditary factors have been shown [46]. Over the past few years, investigations have also found that the incidence of pancreatic cancer in diabetic patients is markedly higher than that in non-diabetics. There is also a definitive connection between pancreatitis and the incidence of PDA [47]. Inflammasome-mediated inflammation is becoming increasingly important in pancreatitis [48]. NLRP3 inflammasomes are closely related to the proliferation of cancer. In related studies, investigators followed several pancreatic cancer patients and assessed their NLRP3 expression using immunohistochemical techniques, in addition to assessing the relationship between survival and NLRP3 expression in these patients by using Kaplan–Meier curves [49, 50]. The results showed that a large proportion of the study population had high expression of the NLRP3 component and that there was an inverse relationship between NLRP3 expression and patient survival. High levels of NLRP3 expression were related to the poor survival rates and bad prognosis. Platelet NLRP3 inflammasomes have been shown to promote platelet aggregation [51, 52]. In a study by Boone et al., platelet NLRP3 inflammasomes were revealed to be upregulated in PDA in a tumor mouse model, further identifying a key role in platelet NLRP3 signaling promoting platelet activation and aggregation in PDA, promoting tumor growth and interfering with tumor survival [53]. Furthermore, interference with platelet NLRP3 signal transduction significantly improves the survival rate of tumor mice. Therefore, platelet NLRP3 inflammasomes play a key role in PDA, which may provide a new treatment method (Fig. 6).

Ductal adenocarcinoma of the pancreas (PDAC) is the most usual kind of pancreatic cancer. It is a highly malignant tumor because of its characteristics of late diagnosis and early metastasis. Gu et al. studied the invasion and migration ability of cells using a Transwell cell invasion test and cell migration test. It was concluded that the depletion of NLRP3 significantly inhibited the tumor progress in PDAC mouse model and obviously decreased the occurrence of lung metastasis, thus revealing that the activation of NLRP3 in tumor-related macrophages enhanced the lung metastasis of pancreatic ductal adenocarcinoma [54].

Data from Daley et al. showed that NLRP3 deletion delayed the malignant progression of pancreatic cancer, was protective against PDA, and NLRP3 signaling promoted accelerated progression of pancreatic tumors [55]. So targeting NLRP3 may have therapeutic promise. Long noncoding RNA (lncRNAs) play an important role in the development and progression of PDA and various tumors. LncRNA microarrays were used to identify new downregulated lncRNAs in cancer tissue, known as XLOC_ 000647. Some results showed that expression of XLOC_000647 was decreased in PDA tissues and cell lines. The expression level of XLOC_000647 was related to tumor stage, lymph node transfer, and overall survival rate [56]. Its high expression level did not only weaken cell multiplication, migration, and epithelial–mesenchymal transition in externa, but also destroyed tumor growth in vivo. In addition, XLOC_000647 can reduce NLRP3 by curbing its promoter. Knockout of NLRP3 can inhibit the proliferation, invasion, and EMT of cancer cells in vitro. In summary, the results of this study suggest that XLOC_000647 not only acts as a new tumor-inhibiting factor of lncRNA, but also may be regarded as a major adjustment factor of NLRP3, which is able to inhibit cell proliferation, invasion, and EMT of PDA. Another study demonstrated that LPS-induced inflammation can activate NLRP3 in the presence of ATP, while NLRP3 would then increase the propagation of pancreatic cancer cells by increasing the activity of caspase-1, causing production of IL-1β [57]. The specific NLRP3 antagonist MCC950 can repress the activation of NLRP3 inflammasomes, which can cut down the vitality of cancer cells. However, the effectiveness of MCC950 varies with cell types. Similarly, 3,4-methylenedioxy-β-nitrophenylethylene (MNS), as a particular NLRP3 inflammasome inhibitor, can repress the expression of NLRP3 inflammasome in cell lines, and NLRP3 inhibition can reduce the proliferation and movement of cancer cells [58].

As mentioned above, IL-1 family cytokines have pleiotropic functions and participate in many inflammatory diseases. IL-18 and IL-1β are members of IL-1 cytokine family. They are important mediators of inflammatory diseases and play a key role in infection and cancer [59]. Although both are activated by inflammatory bodies, there are differences in their regulation. Zaki et al. [60] observed unique expression patterns of IL-18 and IL-1β in the intestine. IL-18 is highly expressed in the colon and provides anti-inflammatory protection by promoting epithelial cell proliferation and tissue repair. In contrast, the basic expression level of IL-1β is low but is enhanced during acute inflammation. Zhu et al. [61] found that the expressions of IL-18 and IL-1β are differentially regulated after LPS stimulation. In the process of chronic stimulation, IL-18 expression is induced and sustained, while that of IL-1β is strongly induced but does not last. Their research also demonstrated that type I IFN signaling is necessary to induce IL-18, but not IL-1β, which indicates that type I IFN plays a key and different role in regulating IL-18 signaling. IL-1 family is induced in tissues immediately after infection and injury, which acts as a key innate immune signal to trigger inflammation. Tumor occurrence and metastasis are related to persistent tissue injury and wound healing, which leads to inflammatory processes involving IL-1 family. Therefore, there is ample evidence that IL-1 family cytokines promote the occurrence and development of cancer [62].

In addition to the above-mentioned effects of activation and inhibition of inflammatory corpuscles on tumorigenesis, gene mutation encoding the inflammatory corpuscles complex also affects tumorigenesis [63]. Ungerbäck et al. [64] showed that the genetic variation of NLRP3 inflammatory body signal transduction-related genes may change the susceptibility and outcome of CRC. Meng et al. [65] found that the missense mutation of NLRP3 coding gene is related to autoimmune diseases characterized by excessive production of IL-1β. The NLRP3 mutation induces the over-activation of inflammatory corpuscles, which leads to immunopathology dominated by Th17 cells in self-inflammation.

### Summary and future prospects

As described above, NLRP3 inflammasomes are an important component of the innate immune system. NLRP3 inflammasomes can be activated by a variety of pathogenic microorganisms and endogenous molecules, resulting in upregulation of IL-1β expression in tissues. The subsequent inflammatory reaction plays a definitive role in the expansion of digestive system cancers. In colorectal cancer, the role of NLRP3 remains controversial, in terms of both protection against tumorigenesis and promoting colorectal carcinogenesis by causing tumor cells to migrate faster. In contrast, in gastric, hepatocellular, and pancreatic cancers, research has confirmed that the stimulation of NLRP3 inflammasomes promotes the invasion and metastasis of cancer cells. In addition, the opportunity to use inflammasome NLRP3 as a therapeutic target for GI cancers and its future therapeutic prospects are greatly increased.

Although we learned that NLRP3 plays a key part in the progression of digestive system diseases, there are still related mechanisms that remain undefined. The mechanism of action of NLRP3 in colorectal cancer is still disputable. In the future, we want to continue to elucidate the mechanism of action and outcome of NLRP3 in colorectal cancer and determine more mechanisms of action and signal transduction pathways between NLRP3 and other digestive system diseases. We will investigate and determine more ways to help a greater number of patients using NLRP3, an inflammatory body, as a therapeutic target for clinical intervention.
